# Understanding the influences and impact of patient‐clinician communication in cancer care

**DOI:** 10.1111/hex.12579

**Published:** 2017-06-21

**Authors:** Jennifer Elston Lafata, Laura A. Shay, Jodi M. Winship

**Affiliations:** ^1^ UNC Lineberger Comprehensive Cancer Center and UNC Eshelman School of Pharmacy The University of North Carolina Chapel Hill NC USA; ^2^ Texas Medical Center University of Texas Houston TX USA; ^3^ School of Medicine Virginia Commonwealth University Richmond VA USA

**Keywords:** cancer care, cancer screening, patient outcomes, patient‐physician communication

## Abstract

**Background:**

Patient‐clinician communication is thought to be central to care outcomes, but when and how communication affects patient outcomes is not well understood.

**Objective:**

We propose a conceptual model and classification framework upon which the empirical evidence base for the impact of patient‐clinician communication can be summarized and further built.

**Design:**

We use the proposed model and framework to summarize findings from two recent systematic reviews, one evaluating the use of shared decision making (SDM) on cancer care outcomes and the other evaluating the role of physician recommendation in cancer screening use.

**Key results:**

Using this approach, we identified clusters of studies with positive findings, including those relying on the measurement of SDM from the patients’ perspective and affective‐cognitive outcomes, particularly in the context of surgical treatment decision making. We also identify important gaps in the literature, including the role of SDM in post‐surgical treatment and end‐of‐life care decisions, and those specifying particular physician communication strategies when recommending cancer screening.

**Conclusions:**

Transparent linkages between key conceptual domains and the influence of methodological approaches on observed patient outcomes are needed to advance our understanding of how and when patient‐clinician communication influences patient outcomes. The proposed conceptual model and classification framework can be used to facilitate the translation of empirical evidence into practice and to identify critical gaps in knowledge regarding how and when patient‐clinician communication impacts care outcomes in the context of cancer and health care more broadly.

## 1 INTRODUCTION

In 2001, the Institute of Medicine issued its landmark *Crossing the Quality Chasm* report which, among other things, called for care to be patient‐centred and based on continuous healing relationships.^1^ High‐quality communication between patients and clinicians is thought to be central to patient‐centred care.^2^, ^3^ Medical payment programmes, including those of the Centers for Medicare and Medicaid Services (CMS), now consider patients’ satisfaction with clinician communication given its importance to care outcomes.^4^, ^5^, ^6^ Despite such endorsements and incentives, how and when different communication exchanges impact specific patient outcomes is only beginning to be understood.

As a body of empirical evidence emerges, a framework is needed to facilitate the understanding of what constitutes effective patient‐clinician communication in given contexts. Early ecological models of patient‐clinician communication often did not consider the diversity of patient outcomes that communication may impact (see for example, Street;^7^ Feldman‐Stewart et al.^8^), or were void of environmental and contextual considerations (see for example Street et al.;^5^ Kreps et al.^9^). Alternatively, models that have more broadly focused on care quality and which consider not only a diversity of patient outcomes, but the complexity of environmental or contextual factors that can impact care processes, including communication, fail to address the complexities and nuances specific to patient‐clinician communication (see for example, Zapka et al.;^10^ Wagner's chronic care model^11^). Thus, while a number of thoughtful models and frameworks are available, none, on its own, can be used to guide a systematic summary of existing empirical evidence on patient‐clinician communication and its impact on patient outcomes or to identify critical gaps in existing knowledge.

In this study, we propose a conceptual model and classification framework upon which the empirical evidence base for the impact of patient‐clinician communication on patient outcomes can be summarized and further built. To illustrate the usefulness of the proposed classification system, we use the approach to summarize findings from two recent systematic reviews of the impact of patient‐clinician communication and patient outcomes^12^, ^13^ focusing on cancer‐related studies.

## 2 COMMUNICATION‐OUTCOMES MODEL

As originally proposed by the Transformation Model of Communication and Health Outcomes (Transformation Model), if communication is an essential process in promoting effective health care, one should be able to demonstrate how communication impacts health and other outcomes.^9^ Our proposed model, therefore like the Transformation Model, has its origins in the systems theory model of input‐process‐output.

**Figure 1:**
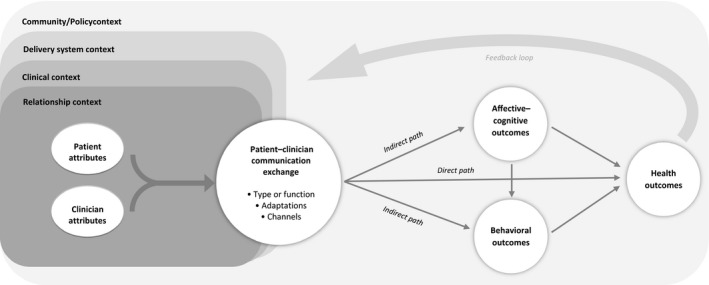
Patient‐clinician communication model

As depicted in Figure 1, at the centre of the model is the patient‐clinician communication exchange. Under the umbrella of high‐quality patient‐clinician communication, there are many types of communication exchanges patients and clinicians may use, ranging from specific communication strategies such as action planning, informed decision making or shared decision making (SDM) to specific functions such as information exchange, question asking or rapport building. These communication exchanges can (but may not) be adapted to fit the needs or preferences of the patient (eg, health literacy levels or language preference). Additionally, these exchanges may occur through a variety of channels (eg, face‐to‐face, telephone or email).

To the left of the communication exchange are the antecedent contextual conditions and attributes patients and clinicians bring with them to the exchange. These include characteristics that can alter the exchange itself, individuals’ interpretation of the exchange or the impact of the exchange on subsequent patient outcomes.^14^ Patient socio‐demographic (eg, age, race, income, and education level) and other characteristics (eg, self‐efficacy, health insurance status) as well as characteristics of the clinician (eg, age, race, medical specialty), are known to be associated with differences in the occurrence of different types of communication exchanges and may also moderate the effect of communication on subsequent outcomes.^15^, ^16^, ^17^, ^18^ In particular, race, gender and age concordance between patients and clinicians has been shown to impact both the communication exchange itself and associated patient outcomes.^19^, ^20^, ^21^

In most cases, communication between patients and clinicians is not limited to a single encounter. Rather, patients form relationships with their clinician(s) over time and often discuss the same health or other topics repeatedly over time. Thus, the length and quality of the emotional and other relationship between the patient and clinician represents one of the first layers of contextual factors that may impact the association between a specific communication exchange and a subsequent patient outcome.^22^, ^23^, ^24^ Following other ecologically based models,^10^ the model highlights not only this relationship context, but also the clinical and delivery system contexts as well as the wider community context. Such an acknowledgement is important as patient‐clinician communication exchanges are likely quite different depending on the disease or treatment being discussed as well as the specific objective of the interaction (eg, initial diagnostic discussion between an oncologist and a woman with early stage breast cancer vs a discussion regarding surgical treatment options between the same woman and a surgeon). Examples of delivery system contextual factors that could influence the communication exchange and its impact on outcomes include the professional and leadership culture of the organization, available resources and procedures within the specific practice setting, and care delivery/management policies. At the community/policy level, the communication exchange and outcomes may be affected by public policy or regulation, professional standards and/or market pressures.^10^ For example, CMS recently issued a requirement for the use of SDM as a prerequisite for lung cancer screening reimbursement.^25^

To the right of the patient‐clinician communication exchange is a range of patient outcomes. Similar to the Transformation Model,^9^ the model conceptualizes outcomes in three broad domains: affective‐cognitive outcomes; behavioural outcomes; and health outcomes.^12^ Affective‐cognitive outcomes include a patient's understanding, knowledge, satisfaction, self‐efficacy, anxieties and the like. Behavioural outcomes encompass a patient's adherence and service use as well as their engagement in other health‐related behaviours (such as diet and physical activities). Health outcomes include morbidity, mortality, quality of life and other related concepts. Despite the wide range of outcomes considered within the scientific literature, there remains little consensus as to the most appropriate or important outcomes to consider when studying the impact of patient‐clinician communication. Thus, like Street and colleagues have proposed,^5^ the model acknowledges that while communication between clinicians and patients can directly lead to improved health outcomes, in most cases, communication affects health indirectly through intermediate outcomes, including affective‐cognitive and behavioural outcomes. Also depicted in the model is a feedback loop that illustrates how patient outcomes impact the subsequent antecedent characteristics of the patient as well as the relational and even broader contexts of subsequent communication exchanges.

## 3 COMMUNICATION‐OUTCOME CLASSIFICATION FRAMEWORK

**Figure 2:**
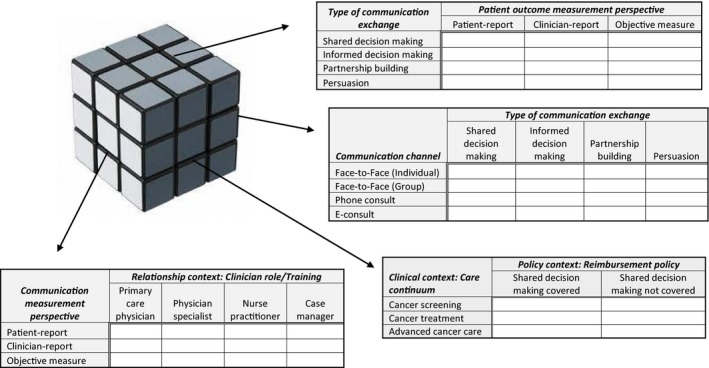
Examples of the communication‐outcome classification framework

While the model depicted in Figure 1 provides a comprehensive conceptualization of how and in what contexts patient‐clinician communication can impact patient outcomes, on its own, it does not offer guidance when synthesizing results across diverse studies or identifying gaps in the existing evidence base. In addition to the conceptual model, we therefore propose a classification framework for organizing results across studies. This requires specification of the types of communication exchange(s) and channel(s) being studied as well as consideration of how each of those may have been adapted to meet patient needs and preferences. It also requires consideration of the specific outcome(s) evaluated and how both the communication exchanges and those outcome(s) are measured. As illustrated in Figure 2, given the multitude of factors that can impact the communication exchange and its impact on patient outcomes, we recommend the use of multiway classification tables. Similar to a Rubik's Cube^®^(Rubiks Brand Ltd, London, England), where algorithms can be used to rotate the blocks to achieve the desired solution, the purposeful selection and organization of key elements can result in the identification of important patterns. As such, the flexibility of a classification table approach affords a practical and transparent visualization of results across diverse studies.

As with a Rubik's Cube^®^, any one side (or classification table) can be displayed in multiple ways. In Figure 2, we have chosen four examples of classification tables that highlight a variety of configurations of factors from our model, including communication exchanges, patient outcomes, measurement perspectives, and contextual factors. Displaying these factors in different configurations allows research questions to emerge around the differences in impact on patient outcomes across factors, combinations of factors that may be driving overall findings across studies, and gaps in the literature. Table 1 provides some examples of research questions that could be associated with the classification tables shown in Figure 2.

**Table 1:** Example research questions to accompany Figure 2 classification tables

1. How does the perspective from which patient outcomes are measured alter the relationship between patient outcomes and different types of communication exchanges?
2. Does the impact of different types of communication exchanges differ by the communication channel used? Does one communication channel seem to better support the use of shared decision making (SDM)?
3. What type of clinical care is now subject to insurance coverage policies targeting patient‐provider communication behaviours? What is the impact of insurers mandating the use of SDM and how might this vary across the cancer care continuum?
4. Are there gaps in the literature regarding the role of nurse communication or the communication of other clinical training on patient outcomes? What communication measurement perspective has been used when studying the role of diverse clinical provider communication on patient outcomes?

Many different types of communication exchanges occur between patients and clinicians and therefore many different types of communication exchanges can be, and are, evaluated by communication and decision scientists. While these different types of exchanges often are compared under a singular heading such as “patient‐centred communication,” such global headings are often not informative as they lack specificity and thus the ability either to replicate findings, or use them to develop communication skills training modules or otherwise inform practice change recommendations. Instead, as depicted in Figure 2, specificity regarding the type of communication exchange is often important. In these cases, not only should the communication exchange be clearly identified and defined, but often equally important is transparency regarding how the specific exchange(s) of interest was measured. Likewise, transparency is needed in how the communication exchange was adapted for individual or population preferences and needs. For example, whether language used by the clinician accounts a patient's health literacy level or language preference likely impacts the exchange content itself and subsequent patient outcomes.

When the patient‐clinician communication is explicitly measured within the context of a study, it is typically measured in one of three ways: patient self‐report, clinician self‐report or observer rating. Even within each of these different measurement perspectives, there are multiple measurement instruments available, often with no agreed upon gold standard.^26^ For example, there are a variety of measures used to ascertain patient‐reported assessments of SDM.^27^, ^28^, ^29^, ^30^, ^31^, ^32^, ^33^ Likewise, there are multiple coding systems available to obtain observer‐rated measures of SDM.^34^, ^35^, ^36^, ^37^ Further complicating matters, patient self‐reports of SDM are often not associated with observer ratings of SDM.^38^, ^39^, ^40^ Thus, as illustrated in Figure 2, the measurement perspective (and instrument used to measure the communication exchange) may be an important factor to consider when evaluating whether and how communication processes impact patient outcomes. It is important to note, that interventional studies may not explicitly measure the communication exchange. Instead, these studies often assume that the presence of an intervention (eg, a decision aid or communication skills training programme) resulted in the use of a particular type of communication exchange (eg, SDM or informed decision making). While such studies enhance our understanding of the impact of a given intervention, without measurement of the communication exchange(s), they do not directly enhance our understanding of the impact of a specific type of communication exchange.

Another, increasingly important consideration is the channel of communication. The number and variety of channels through which clinicians and patients are communicating today is unprecedented. Studies have examined a wide range of innovative communication channels beyond traditional face‐to‐face interactions including text messaging,^41^ e‐mail,^42^ video‐based telemedicine^43^ and even social media such as Twitter and Facebook.^44^ As of yet, limited research has explored how these channels alter patient‐clinician communication exchanges or alter the outcomes associated with those exchanges.

Specificity is also important in the type of patient outcome(s) considered. While some of the cognitive‐affective outcomes (eg, satisfaction and trust) are by definition patient‐reported measures, behavioural and health‐related outcomes may be measured either by patient or clinician self‐report, or by more objective measurement methods. For example, a patient could be asked to rate their current stress level or their stress could be measured via salivary cortisol—neither of which has been found to correlate highly with one another^45^ and each of which may be worthy of consideration within a specific study context. As the effect of patient‐clinician communication on patient outcomes may vary across outcomes and how those outcomes are measured, transparency in outcomes and measurement methods is also needed.

## 4 APPLICATION OF MODEL AND FRAMEWORK TO SHARED DECISION‐MAKING COMMUNICATION

Using the conceptual model, combined with the classification framework, enables not only the ability to transparently summarize the impact of communication exchanges on diverse patient outcomes, but also the ability to identify knowledge gaps where subsequent research is needed. For example, we recently applied this approach to summarize the evidence for the effect of SDM on patient outcomes.^12^ In that application, we held the communication exchange type constant (ie, SDM), but considered the different types of outcomes (ie, affective‐cognitive, behavioural, or health outcome) that had been studied in relationship with SDM as well as the different perspectives from which the measurement of SDM had occurred.^12^ In so doing, we were able to highlight the importance of the communication measurement perspective used, finding that SDM, as reported by patients as occurring, was associated with improvements in affective, and in some cases, behavioural outcomes. Table 2 reports findings from the 48 cancer specific studies included in that review. As illustrated in Table 2, similar clusters of studies with positive findings can be seen among the cancer‐specific studies, including those relying on the measurement of SDM from the patients’ perspective and affective‐cognitive outcomes. Also illustrated is the void in studies that have considered clinician perceptions of SDM as well as those evaluating the impact of SDM patient on health outcomes.

**Table 2:** Summary of results by measurement perspective and patient outcome category: impact of shared decision making on patient outcomes

Measurement perspective	Results	Patient outcome category							
		Affective‐cognitive		Behavioural		Health		Total	
		n	%	n	%	n	%	n	%
Patient reported	Positive	8	50	3	30	1	9	12	32
	NS	6	38	7	70	10	91	23	62
	Negative	2	13	0	0	0	0	2	5
	Total measured	16		10		11		37	
Clinician reported	Positive	0	–	0	–	0	–	0	0
	NS	0	–	0	–	0	–	0	0
	Negative	0	–	0	–	0	–	0	0
	Total measured	0	–	0	–	0	–	0	0
Observer rated	Positive	2	22	0	0	0	–	2	18
	NS	7	78	2	100	0	–	9	82
	Negative	0	0	0	0	0	–	0	0
	Total measured	9		2		0	–	11	
Total	Positive	10	40	3	25	1	9	14	29
	NS	13	52	9	75	10	91	32	32
	Negative	2	8	0	0	0	0	2	4
	Total measured	25		12		11		48	

Adapted from Shay & Elston Lafata^12^.

NS, NonSignificant.

We further illustrate the usefulness of the model and classification framework by using it to consider findings from the same systematic review albeit from a different perspective.^13^ In this second example, (Table 3) we evaluate the association between SDM and different types of patient outcomes (affective‐cognitive, behavioural, and health) by the specific clinical decision context (eg, surgical treatment, treatment [general], post‐surgery/adjuvant therapy, and end‐of‐life care). Examining the same studies using different dimensions provides additional information, allowing for more nuanced conclusions. In particular, this rotation highlights the fact that the role of SDM in cancer care is most often studied in the context of surgical decision making, and rarely in the context of post‐surgical treatment or end‐of‐life care. This rotation further reinforces that positive relationships between SDM and patient outcomes, when detected, tend to be among affective‐cognitive outcomes, most often in the context of surgical treatment decisions.

**Table 3:** Summary of results by decision type and patient outcome category: impact of shared decision making on patient outcomes

Patient outcome category	Results	Clinical context: type of decision									
		Surgical treatment		Treatment (General)		Post‐surgery/adjuvant		End‐of‐life care		Total	
		n	%	n	%	n	%	n	%	n	%
Affective‐cognitive	Positive	6	50	4	36	0	0	0	–	10	40
	NS	4	33	7	64	2	100	0	–	13	52
	Negative	2	17	0	0	0	0	0	–	2	8
	Total measured	12		11		2		0		25	
Behavioural	Positive	1	13	1	33	1	100	0	–	3	25
	NS	7	88	2	67	0	0	0	–	9	75
	Negative	0	0	0	0	0	0	0	–	0	0
	Total measured	8		3		1		0		12	
Health	Positive	1	25	0	0	0	–	0	0	1	9
	NS	3	75	3	100	0	–	4	100	10	91
	Negative	0	0	0	0	0	–	0	0	0	0
	Total measured	4		3		0		4		11	
Total	Positive	8	33	5	29	1	33	0	0	14	29
	NS	14	58	12	71	2	67	4	100	32	32
	Negative	2	8	0	0	0	100	0	0	2	4
	Total measured	24		17		3		4		48	

Adapted from Shay & Elston Lafata^12^.

NS, NonSignificant.

Finally, using findings from a recently published systematic review of the impact of provider‐patient communication on cancer screening adherence,^13^ we illustrate the importance of specificity regarding the type of communication exchange, particularly to identify gaps in the literature. As depicted in Table 4, while a number of studies have considered the impact of physician recommendation on patients’ adherence to cancer screening recommendations, no other specific communication exchange type has been considered across multiple studies, thus limiting our ability to draw conclusions about the impact of specific physician communication strategies when recommending cancer screening to their patients.

**Table 4:** Summary of results by communication exchange type and cancer type: impact of patient‐clinician communication on screening use

Clinical context: type of cancer	Results	Communication exchange type													
		Recommendation		Informed Decision Making		5As		Persuasion		Enthusiasm		Explaining/Counselling		Total	
		n	%	n	%	n	%	n	%	n	%	n	%	n	%
Cervical cancer screening	Positive	5	100	0	–	0	–	0	–	0	–	1	100	6	100
	NS	0	0	0	–	0	–	0	–	0	–	0	0	0	0
	Positive+Negative	0	0	0	–	0	–	0	–	0	–	0	0	0	0
	Total measured	5		0		0		0		0		1		6	
Colorectal cancer screening	Positive	14	100	1	50	1	100	0	0	0	–	1	100	17	89
	NS	0	0	0	0	0	0	1	100	0	–	0	0	1	5
	Positive+Negative	0	0	1	50	0	0	0	0	0	–	0	0	1	5
	Total measured	14		2		1		1		0		1		19	
Breast cancer screening	Positive	4	100	0	–	0	–	0	–	1	100	2	100	7	100
	NS	0	0	0	–	0	–	0	–	0	0	0	0	0	0
	Positive+Negative	0	0	0	–	0	–	0	–	0	0	0	0	0	0
	Total measured	4		0		0		0		1		2		7	
Total	Positive	23	100	1	50	1	100	0	0	1	100	3	100	29	91
	NS	0	0	0	0	0	0	1	100	0	0	0	0	1	3
	Positive+Negative	0	0	1	50	0	0	0	0	0	0	0	0	1	3
	Total measured	23		2		1		1		1		3		32	

Adapted from Peterson et al., Tables 2 and 3;^13^ excludes Mah & Bryant^48^ due to lack of statistical testing.

NS, NonSignificant.

## 5 DISCUSSION

We propose the use of a conceptual model and framework to consider the impact of patient‐clinician communication on cancer care outcomes. The model highlights the complexity of the relationship between patient‐clinician communication and patient outcomes, the diversity of communication exchanges that transpire between patients and clinicians, the diversity of channels now used by clinicians and patients for communication exchanges, and the multilevels of contextual factors that can influence patient‐clinician communication and its impact. Permutations of the multiway classification tables highlight the importance of methodological choices, and the need for specification of patient outcome, communication exchange, and clinical decision context when considering the impact of patient‐clinician communication in the context of cancer care. Using this approach, we identified clusters of studies with positive findings, including those relying on the measurement of SDM from the patients’ perspective and affective‐cognitive outcomes, particularly in the context of surgical treatment decision making. We also identify important gaps in the literature, including the role of SDM in post‐surgical treatment and end‐of‐life care decisions, and those specifying particular physician communication strategies when recommending cancer screening.

Particularly unique to our model is the emphasis on the context in which the communication occurs. This includes not only the communication channel, but also things like the patient‐provider relationship context as well as the broader organizational and policy context. It is our belief, that just as the importance of context is beginning to be understood in the field of implementation science, so too is the importance of context in the communication field likely to grow.^6^ This is due in part to the increased attention and weight patient‐communication is receiving within the realm of pay‐for‐performance plans. It is also due to our increased understanding of the importance of social factors as health determinants. As payment and other performance monitoring programmes enhance their focus on clinician communication or even mandate the use of specific types of communication exchanges (as Medicare has recently done for SDM in the context of lung cancer screening^25^) opportunities to study the role of context on communication and its impact on patient outcomes is likely to increase.

The challenges faced in improving patient‐clinician communication have been well documented.^46^ Those commonly mentioned include time constraints, a lack of clinician training, and perverse financial incentives. Less commonly mentioned are the voids in understanding which types of communication impact which types of outcomes. In fact, while there have been a number of trials examining the effects of interventions designed to alter patient‐clinician communication, as well as a number of observational studies assessing the association between patient‐clinician communication and patient outcomes, results from these studies are often inconsistent.^12^, ^47^ Both conceptual and methodological differences likely contribute to these inconsistencies. Prior studies of patient‐clinician communication in the context of cancer care have considered a diversity of communication exchanges, which have been measured from an array of perspectives. Furthermore, the communication exchanges studied are assessed in relation to a similarly diverse array of patient outcomes that are likewise measured from a multitude of perspectives and via multiple approaches. Such methodological differences present challenges for combining results across studies—whether systematically or otherwise.^26^

By using the model and framework proposed here, we have illustrated the importance of the measurement perspective used to assess the communication exchange as well as the type of outcome considered when evaluating the impact of SDM.^12^ In the application here, we further demonstrate how this approach can be used to explore multiple dimensions within an existing evidence base to highlight important gaps and methodological variability. Despite this ability, it nonetheless is important to note that the model and accompanying framework approach remain a simplification. As such they may omit from consideration other important factors that impact either the communication exchange itself or its impact on outcomes. Furthermore, it is important to acknowledge challenges have and will continue to exist in finding or constructing databases that enable any individual study to encompass all the components of the model presented here.

It is now accepted that patient‐clinician communication can and does impact patient outcomes. Using studies from two systematic reviews and the conceptual model and classification framework proposed, we are able to begin to understand how and when patient‐physician communication exchanges impact specific outcomes**.** Our model and classification framework provide a pragmatic depiction of the complex array of factors that can impact patient‐clinician communication exchanges and the impact those communication exchanges have on patient outcomes. While the model and framework alone do not simplify the study of this complex phenomenon, they do offer an organizing structure within which to design, evaluate and discuss patient‐clinician communication studies. The challenge of conducting such studies to understand this diverse and complex phenomenon remains.

## ACKNOWLEDGEMENTS

Dr. Lafata is supported by the National Cancer Institute at the National Institutes of Health (R01 7R01CA197205‐02). Dr. Shay was supported by the National Cancer Institute at the National Institutes of Health (R25 CA57712).

## CONFLICTS OF INTEREST

None.
